# Supplementary material for: The Evolving Landscape of Radiomics in Gliomas: Insights into Diagnosis, Prognosis, and Research Trends
Source: Cancers (Basel). 2025 May 6;17(9):1582. doi: 10.3390/cancers17091582 (PMC12071695; doi:10.3390/cancers17091582)
Supplement: Supplementary file 1 [file cancers-17-01582-s001.zip › cancers-3607592-supplementary.pdf]

Supplementary Table S1. MRI Sequence Utilized by Study Type

Author	Year	T1w	T1wCE	T2w	FLAIR	DTI
**Glioma Differential Diagnosis (n=13)**						
Bai et al.	2024	1	1	1	1	0
Bathla et al.	2024	1	1	1	1	1
Battalapalli et al.	2024	0	1	1	1	0
Li et al.	2024	0	1	0	0	0
Lin et al.	2024	1	1	1	1	1
Wang et al.	2024	1	1	1	1	1
Zhang et al.	2024	0	1	1	1	0
Biggs et al.	2023	1	1	1	1	1
Felefly et al.	2023	0	1	0	0	0
Shao et al.	2023	1	1	1	1	1
Tippareddy et al.	2023	1	1	1	1	0
Yan et al.	2023	1	1	1	1	1
Yu et al.	2023	0	1	0	1	0
Zhang et al.	2023	1	1	1	0	1
Total		8	13	10	10	6
**Outcome Prediction (n = 11)**						
Gao et al.	2024	1	1	1	1	0
Karabacak et al.	2024	1	1	1	1	0
Liu et al.	2024	1	1	1	1	0
Zhang et al.	2024	1	1	1	1	0
Bathla et al.	2023	1	1	1	1	1
GloryPrecious et al.	2023	0	1	0	0	0
Hajianfar et al.	2023	0	1	0	1	0
Joo et al.	2023	1	1	1	1	0
Kaur et al.	2023	1	1	1	1	0

Yousaf et al.	2023	1	1	1	1	0
Yun et al.	2023	0	0	0	0	1
Total		8	10	8	9	2
**Feature Extraction/Segmentation (n = 8)**						
Beser-Robles et al.	2024	0	1	1	1	0
Falco-Roget et al.	2024	1	0	0	0	0
He et al.	2024	1	1	1	1	1
Muthusivarajan et al.	2024	1	1	1	1	0
Vossough et al.	2024	1	1	1	1	0
Bhattacharjee et al.	2023	1	0	1	1	1
Chilaca-Rosas et al.	2023	0	1	1	0	0
Papi et al.	2023	1	1	1	1	0
Parvaze et al.	2023	1	0	1	1	1
Total		7	5	7	7	3
**Glioma Grading (n = 6)[a]**						
Lin et al.	2024	1	1	1	1	0
Sun et al.	2024	1	1	1	1	0
Renugadevi et al.	2023	1	1	1	1	0
Ubaldi et al.	2023	1	1	1	1	0
Zhu et al.	2023	0	1	0	0	0
Total		4	5	4	4	0
**Other (n = 4)[b]**						
Kwak et al.	2024	1	1	1	1	1
Danilov et al.	2023	1	1	1	1	1
Nalepa et al.	2023	1	1	1	1	0
Total		3	3	3	3	2

[a]Maskani et al. utilized CT imaging
[b]Danilov et al. utilized PET/CT imaging
